# Longitudinal blood gas profiling reveals relative systemic hypoxia and its consequences for health and production in transition dairy cows

**DOI:** 10.5713/ab.250687

**Published:** 2026-06-01

**Authors:** Fangchao Wu, Feixiang Fan, Zhiyao Guo, Haoyu Che, Zihai Wei, Hong-Yun Liu, Hui-Zeng Sun, Jian-Xin Liu, Feng-Fei Gu

**Affiliations:** 1 Institute of Dairy Science, College of Animal Sciences, Zhejiang University, Hangzhou, China; 2 College of Animal Sciences, Xinjiang Key Laboratory of Herbivorous Nutrition for Meat & Milk, Xinjiang Agricultural University, Urumqi, China; 3 Ministry of Agriculture and Rural Affairs Key Laboratory of Dairy Cattles Genetic Improvement in Southern China, Bright Farming Co., Ltd., Shanghai, China

**Keywords:** Dairy Cow Health, Hypoxia, Hypoxic Stress, Peripartum Period

## Abstract

**Objective:**

This study aimed to investigate whether transition dairy cows experience a negative oxygen balance due to increased metabolic demands postpartum and to assess how this imbalance affects their health and production performance. The goal was to elucidate the longitudinal changes in blood gas parameters and establish links between hypoxic stress and physiological, inflammatory, and productive indicators.

**Methods:**

Ninety Holstein cows were monitored from 21 days before to 49 days after calving, with blood gas parameters, plasma biochemical indices, dry matter intake, and milk production being recorded at multiple time points. To assess the impact of hypoxia, cows were further grouped by postpartum hypoxia-inducible factor 1α (HIF-1α) levels into high and low hypoxic stress groups for comparative analysis.

**Results:**

During the transition period, dairy cows exhibited a significant postpartum reduction in arterial and venous hemoglobin (Hb), hematocrit (HCT), and oxygen concentration, reaching the lowest levels at 21 days after calving, while CO_2_-related parameters like TCO_2_, HCO_3_^−^, and BEecf increased. Cows with higher HIF-1α levels (HHS group) showed decreased dry matter intake and milk fat concentration, along with higher plasma β-hydroxybutyrate, malondialdehyde, and haptoglobin levels. Furthermore, significant correlations were observed between blood gas indicators and health parameters, including a negative correlation of SAA with Hb and HCT, highlighting the link between impaired oxygen transport and systemic stress responses.

**Conclusion:**

Postpartum dairy cows characterized by decreased oxygen delivery (lower Hb and HCT) and increased oxygen consumption (elevated CO_2_-related parameters), leading to relative metabolic systemic hypoxic stress. This imbalance is associated with liver stress, inflammation, oxidative damage, and reduced feed intake and milk production. These findings underscore the critical need for nutritional and management strategies targeting oxygen availability to support health and productivity during the transition period.

## INTRODUCTION

The peripartum period is a critical stage, referring to the time from three weeks pre-calving to three weeks post-calving [1]. During this period, dairy cows are particularly susceptible to various metabolic disorders such as ketosis, retained placenta, and displaced abomasum as a result of the substantial physiological, environmental, and management changes they encounter [2]. It is reported that 30%–50% of cows experience one or more diseases during this period [3], leading to serious losses in animal welfare and economic profitability. Therefore, ensuring the health of lactating cows during the perinatal period is essential for maximizing their milk-producing potential and improving economic benefits.

Adequate metabolizable oxygen is essential for cellular function and overall physiological homeostasis, playing a critical role in metabolic processes that sustain life [4]. Conversely, insufficient oxygen availability, commonly referred to as hypoxia or hypoxic stress, is characterized by higher expression of hypoxia-inducible factor 1α (HIF-1α) [5], and can adversely affect health and metabolic processes. Previous study reported hypoxia acts as a global regulator, influencing the inflammatory and innate immune functions of macrophages and neutrophils [6]. Recently, it was reported that cattle with higher milk yield had increased blood oxygen concentration and transport ability [7]. In addition, hypoxic conditions can impair the lactation sustainability of dairy cows through the HIF-1α–FOS signaling pathway [8]. Furthermore, hypoxia also hinders the formation of mammary alveoli, subsequently impairing milk secretion capacity [9]. And hypoxia has been identified as a significant contributor to the adverse effects of heat stress on both production performance and health in dairy cows [10]. These studies have shown that reduced metabolizable oxygen availability plays important roles in animal health and production performance.

For the postpartum dairy cows, transiting from the dry period to lactation, experience a sharp rise in metabolic activity and consequently a substantial increase in oxygen demand [11]. As milk production increases, the basal metabolic rate of the cow significantly elevates, with enhanced carbohydrate metabolism to supply the energy needed for high levels of milk synthesis [12]. Concurrently, the periparturient period is often accompanied by a negative energy balance, with increased fat mobilization in dairy cows [3]. Furthermore, the postpartum inflammatory responses activate immune cells, which depend on sufficient oxygen to mount effective responses against pathogens [13,14]. These metabolic and immune processes, especially in metabolically active tissues like the mammary glands and liver, necessitate substantial oxygen to generate ATP [15]. Given these physiological demands, it is speculated that the oxygen demand of postpartum cows is substantially higher, rendering them potentially vulnerable to deficiencies in oxygen supply and resulting in relative hypoxia. However, the determination of whether transitional cows are experiencing systemic hypoxic stress remains to be established. Blood gas parameters serve as reliable indicators of systemic oxygen utilization and metabolic processes [16]; however, their longitudinal changes during the transition period in dairy cows remain poorly characterized.

Therefore, we hypothesized that the increased metabolic demand during the transition period may create a negative oxygen balance in dairy cows, leading to relative hypoxia and consequently affecting their health status and production performance. A longitudinal experiment was designed to investigate whether dairy cows experience oxygen deficiency or hypoxic stress due to increased metabolic demands during the transition period, and to clarify their potential effects on the health and production performance of dairy cows postpartum. Our research provides new insights into the physiological metabolism of dairy cows during the transition period, aiding in the development of strategies to improve the health of transitional dairy cows.

## MATERIALS AND METHODS

### Animals and experimental design

A total of 90 healthy multiparous Chinese Holstein cows were selected at d −28 relative to calving. The experiments commenced 21 days before calving and concluded 49 days post-calving. The health was defined based on veterinary records and diagnoses, with no clinical or subclinical diseases occurring within one month prior to the study. Throughout the trial, cows were housed in individual tie-stalls and were fed three times daily with free access to water. In the first part of the experiment, blood gas parameters were measured in 90 cows at d −21, −7, 0, +7, +21, +35, and +49 to characterize dynamic changes in oxygen metabolism during the transition period. After controlling for calving date (within ±2 d of the expected date), sample completeness, and health status, 46 cows (parity = 2.0±0.5; body condition score [BCS] = 3.0±0.25) remained for the final analysis. The health was defined based on veterinary records and diagnoses, with no clinical or subclinical diseases occurring during the study period. In the second part, to further evaluate the impact of hypoxia, plasma HIF-1α concentrations at d +21 were ranked across these 46 cows. Cows in the top quartile were classified as the high hypoxic stress group, while those in the bottom quartile were classified as the low hypoxic stress group. Sample size was determined using an a priori power analysis conducted in SAS 9.4 (PROC POWER). Variance estimates were derived from preliminary blood gas data collected from a pilot cohort of 46 cows. The calculation was based on plasma HIF-1α concentration data from a pilot study. To detect the anticipated differences with a significance level (α) of 0.05 using a one-tailed t-test, a sample size of 10 cows per group was required to achieve a statistical power of 0.90. Thus, from above quartile ranges, 10 cows with the most extreme values were selected for each group to maximize the contrast between high (HHS) and low (LHS) hypoxic stress. Detailed information on parity, BCS, and milk efficiency for both group cows is presented in Supplement 1.

### Dry matter intake and feed samples analysis

The dry matter intake (DMI) was measured every two weeks from 3 weeks prepartum to 5 weeks postpartum. Measurements were conducted on d 6 and 7 of week, with feed samples collected on d 7. The feed samples were analyzed according to standard methods for nutritional components (AOAC [17]), including DM (105°C for 5 hours), CP (# 988.05), ash (# 942.05), and ADF (# 973.18). Analyses of NDF and starch were performed using the method of Van Soest et al. [18]. The concentrations of phosphorus (P) and calcium (Ca) were measured using inductively coupled plasma emission spectroscopy (Optima 4300DV; Perkin Elmer). The nutritional composition of the experimental diet is shown in Table 1.

### Milk yield and composition analysis

Postpartum milk yield was recorded weekly for weeks 1 to 5. On d 6 and 7 of each week, milk yield was continuously measured using a flow meter (Flowmaster Pro; DeLaval), and milk samples were collected on day 6. Samples were collected in a 50 mL tube at a ratio of morning: noon: evening = 4:3:3, with bronopol tablets added (milk preservative; D and F Control Systems), and stored at 4°C for later analysis. Milk components (fat, protein, lactose, TS, and SCC) were analyzed using infrared spectrophotometry. The ECM (kg/d) = 0.3246× milk yield (kg/d)+13.86×milk fat yield (kg/d)+7.04×milk protein yield (kg/d).

### Blood sample collection and analysis

Blood samples were collected from the coccygeal vein and artery of each cow and deposited into 10 mL tubes containing heparin lithium at d −21, −7, 0, +7, +21, +35 and +49. The blood collection tubes used in this study were disposable vacuum tubes equipped with rubber stoppers. During sampling, the needle was inserted directly into the tube to allow blood to flow immediately under vacuum conditions. Once blood flow ceased, the needle was promptly removed. In addition, multiple punctures of the tube stopper were strictly avoided to prevent potential air contamination. The 200 μL of the blood was added in the i-STAT CG8+cartridge (Abbott Medical) card to measure blood gas parameters with i-STAT1 portable clinical analyzer (#i-STAT1 300G; Heska Corporation) immediately. Each i-STAT blood gas card came with a calibration solution for calibration. The analyzed blood gas parameters including pH, concentrations of sodium (Na^+^), potassium (K^+^), total carbon dioxide (TCO_2_), and ionized calcium (iCa), along with hemoglobin (Hb), oxygen partial pressure (pO_2_), carbon dioxide partial pressure (pCO_2_), bicarbonate (HCO_3_^−^), and excess base in extracellular fluid (BEecf). Furthermore, the blood oxygen concentration was calculated using the following equation [19]:


(1) $$\text{Oxygen concentration }({\text{cO}}_{2},\text{mL}/\text{dL})=0.003\times {\text{pO}}_{2}+1.34\times \text{Hb}\times {\text{sO}}_{2},$$

Where, pO_2_ is the oxygen partial pressure (mmHg), Hb is hemoglobin (g/dL), and sO_2_ is blood oxygen saturation (%). The arteriovenous difference (AVD) of the oxygen concentration was calculated according to reported methods [8].

Additionally, the remaining venous blood samples were centrifuged at 3,000×g for 15 min at 4°C to collect plasma, followed by frozen at −20°C until further analysis. An autoanalyzer 7020 instrument (Hitachi High-Technologies Corporation) was utilized to determine the alanine aminotransferase (ALT, #ZH2001G), aspartate aminotransferase (AST, #ZH2002G), BUN (#ZH2017S), total protein (TP, #ZH2012G), albumin (#ZH2013G), glucose (#ZH2079T), non-esterified fatty acids (NEFA, #ZH2045Z), BHBA (#ZH2029T), triglycerides (#ZH2039Z), cholesterol (#ZH2040Z), superoxide dismutase (SOD, #ZH2058F), glutathione peroxidase (GSH-Px, #A005-1-2), and total antioxidant status (TAS, #ZH2039Z). These biochemical parameters were determined using automated enzymatic based on spectrophotometric detection.

Concentrations of plasma HIF-1α (#307-2), malondialdehyde (MDA, #A003-1-2), total oxidant status (TOS, #KC5100), haptoglobin (HPT, #H136), and serum amyloid A (SAA, #H134) were quantified using commercial assay kits from Nanjing Jiancheng Bioengineering Institute, following the manufacturer’s instructions. Finally, total oxidative status (TOS; #KC5100) was measured according to the manufacturer’s protocol. These assays were primarily based on enzyme-linked immunosorbent assay (ELISA). The oxidative status index (OSI) was defined as the ratio of TOS to TAS [20].

### Statistical analysis

The longitudinal blood gas and plasma parameters were analyzed using the PROC MIXED procedure with repeated measurement in SAS software (ver. 9.4), and covariance type AR (1) was selected. The model included the fixed effect of week and cow within the week as a random effect. The experimental results were reported as least squares means, and the differences were separated using the PDIFF option in the LSMEANS treatment. Phenotypic data and plasma parameters between HHS and LHS groups were statistically analyzed using the mixed-effects model in SAS (ver. 9.4). The analysis was conducted according to a randomized with repeated measurement block design, with group, time, and their interaction (group×time) as fixed effects, and block and cow as a random effect. Results are presented as mean and SEM. p<0.05 indicates that the data are significantly different, and 0.05≤p≤0.10 indicates that the data have a trend of differences.

## RESULTS

### Longitudinal production performance and blood gas changes during the transition period

**Figure 1:**
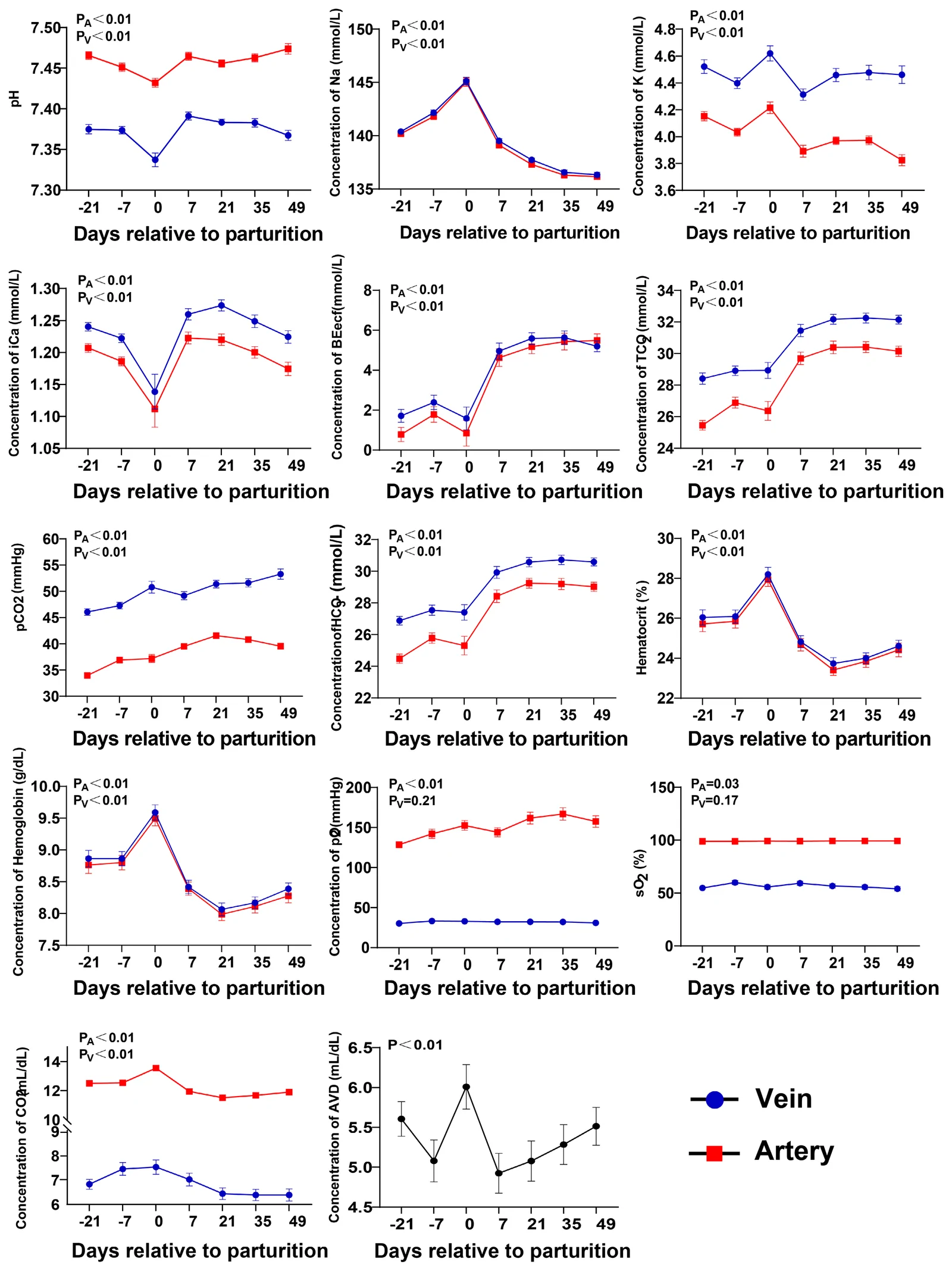
Dynamic changes of blood gas parameters in 46 dairy cows before and after calving at −21 d, −7 d, +7 d, +21 d, +35 d, and +49 d. pO_2_, partial pressure of oxygen; sO_2_, oxygen saturation; cO_2_, concentration of oxygen = 0.003×pO_2_+1.34×Hb×sO_2_; AVD, artery vein difference.

As shown in Figure 1, arterial and venous pH, Na^+^, K^+^, iCa, BEecf, TCO_2,_ pCO_2_, HCO_3_^−^, HCT, Hb, cO_2_ and AVD were all exhibited temporal differences (p<0.01), no significant temporal difference in arterial sO_2_ (p = 0.17) and venous pO_2_ (p = 0.21) were observed, but venous sO_2_ (p = 0.03) and arterial pO_2_ (p<0.01) showed temporal significant differences. In specific, the pH showed a significant decrease trend before delivery and then increased at the first postpartum week and then gradually stabilized. The concentrations of Na+ and K+ peaked at delivery and the iCa was opposite. The BEecf changed slightly in the prenatal period but increased significant postpartum, with a plateau after reaching to the highest point at d +21. The changing trends of TCO_2_ and HCO_3_^−^ were consistent with BEecf. The venous pCO_2_ increased slowly at prepartum, declined in the first week postpartum, and then rose gradually again. Arterial pCO_2_ increased slowly until day 21 postpartum and subsequently decreased. Arterial and venous HCT, Hb and cO_2_ present an increase from d −21 to calving day, but decreased after delivery and reached the lowest point at d +21. Additionally, the AVD reached peak at 0 d, and then increased slowly from d +7 to d +49. The dynamic changes of DMI and milk yield are shown in Supplement 2, which present increasd from d+7 until to the +35 d (p<0.01).

### Correlation analysis of health indicators, production performance, and blood gas parameter at postpartum

**Figure 2:**
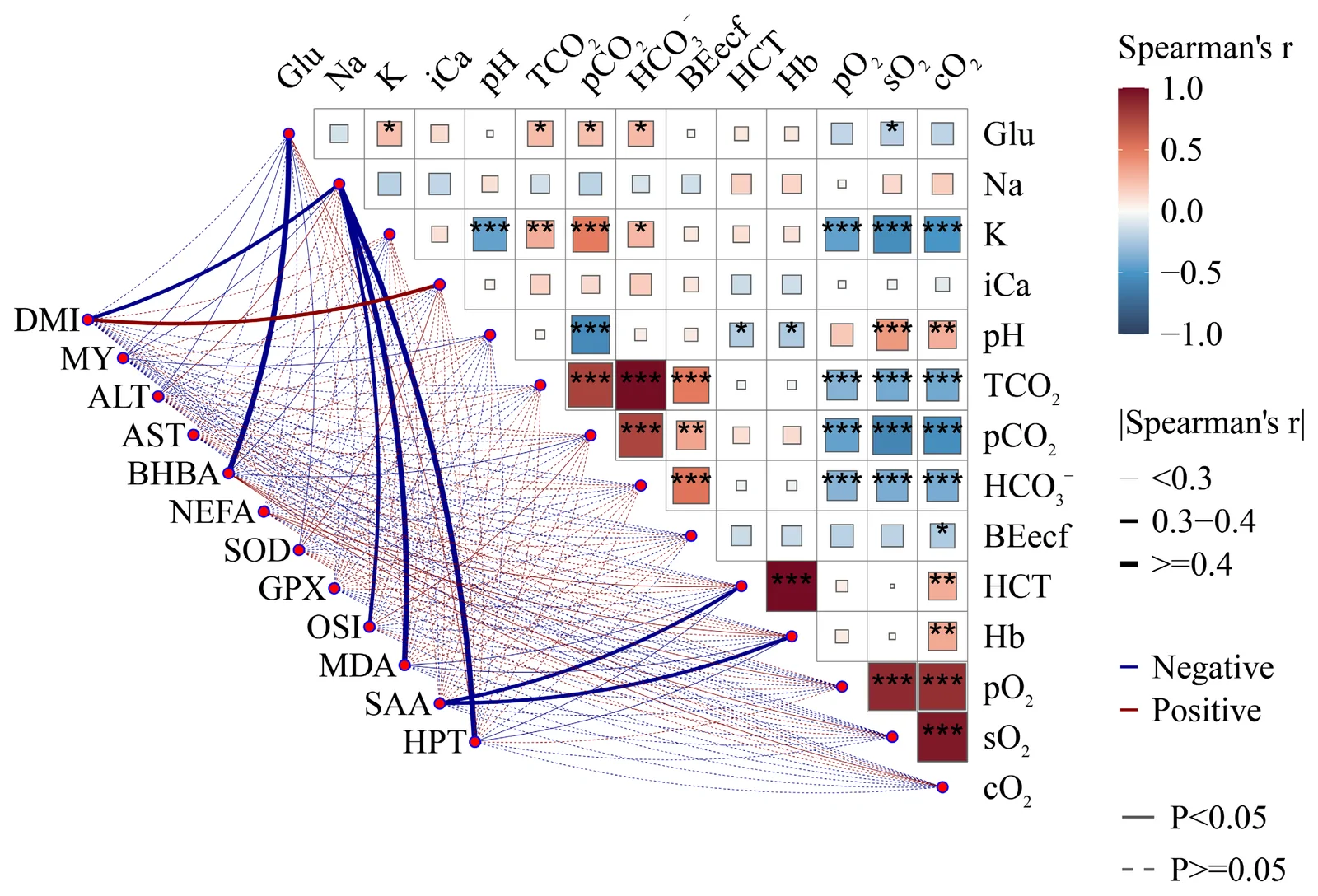
Spearman correlation analysis of bovine tail vein blood gas spectrum with oxidative stress, inflammation, liver function, and productivity at +7 d and +21 d postpartum. Red represents positive correlation, blue represents negative correlation, p<0.05 is a solid line, and p≥0.05 is a dashed line. * p<0.05, ** p<0.01, and *** p<0.001. DMI, dry matter intake; MY, milk yield; ALT, alanine aminotransferase; AST, aspartate aminotransferase; BHBA, β-hydroxybutyrate; NEFA, nonesterified fatty acids; SOD, superoxide dismutase; GPX, glutathione peroxidase; OSI, oxidative stress index; MDA, malondialdehyde; SAA, serum amyloid A; HPT, haptoglobin; pO_2_, partial pressure of oxygen; sO_2_, oxygen saturation; cO_2_, concentration of oxygen = 0.003×pO_2_+1.34×Hb×sO_2_.

The venous plasma physiological parameters are presented in Supplement 3. The correlation between postpartum plasma physiological parameters and blood gas parameters was shown in Figure 2. The DMI (r = −0.39, p<0.01), HPT (r = −0.42, p<0.01), MDA (r = −0.48, p<0.01) and OSI (r = −0.35, p<0.01) showed a significant negative correlation with the concentration of Na+. And DMI (r = 0.31, p<0.01) showed a significant positive correlation with iCa content. Additionally, the SAA showed a negative correlation with HCT (r = −0.33, p<0.01) and Hb (r = −0.31, p<0.01).

### Differences in production performance, blood gas and plasma physiological parameters between HHS and LHS cows

The basic information of the cows in LHS and HHS is presented in Supplement 1. No differences in parity (p = 0.34), milk yield (p = 0.87), lactation efficiency (p = 0.54) and BCS (p = 0.98) between LHS and HHS, only the HIF-1α content (p<0.01) and AVD (p<0.01) in LHS significant lower compared to the HHS cows. The comparison of lactation performance between HHS and LHS cows is presented in Table 2. The postpartum and prepartum DMI in LHS cows was significantly (p = 0.04) higher and tend to be higher (p = 0.10) than these cows in HHS group, respectively. Additionally, the yield of milk (p = 0.07) and ECM (p = 0.09) were tended to be higher in LHS cows. Furthermore, the MUN was significantly higher in the LHS cows than that of HHS cows (p = 0.03).

As showed in Table 3, the concentration of venous HIF-1α is significant higher in HHS group than those in LHS (p<0.01). No significant differences of arterial blood gas parameters between HHS and LHS groups were observed. However, the venous pH (p = 0.02) and pCO_2_ (p<0.01) of the HHS group were significantly lower than that of the LHS group. Conversely, the pO_2_ (p = 0.01), sO_2_ (p<0.01) and cO_2_ (p<0.01) were significantly higher in the HHS group. Furthermore, the pH (p = 0.02), K^+^ (p = 0.01), pO_2_ (p = 0.04) and sO_2_ (p = 0.01) of venous had the effects differed across the time points, while the Na^+^ (p = 0.01) and cO_2_ (p<0.01) of artery had time effects. The venous plasma parameters differences between LHS and HHS are presented in Table 4. Compared with the HHS group, the LHS group had lower concentration in BHBA (p = 0.01) and HPT (p<0.01) and had lower tendency in ALT (p = 0.08), BUN (p = 0.09) and MDA (p = 0.07).

## DISCUSSION

During the periparturient period, dairy cows undergo rapid physiological and metabolic transitions, including fetal growth, parturition, and the onset of lactation, which significantly increase oxygen demand [21]. However, the changes in metabolizable oxygen and their subsequent effects during the transition period remain unclear. In this study, we measured longitudinal blood gas parameters to explore alterations in oxygen metabolism. Postpartum, we observed decreases in parameters related to oxygen supply and transport, including HCT and Hb, indicating a deficiency in metabolizable oxygen reflected by reduced blood oxygen concentration. Furthermore, we found this insufficiency was associated with liver damage, inflammation, and reduced production potential in dairy cows.

The metabolic processes involved in oxidizing energy substrates like glucose and fatty acids require oxygen consumption and generate CO_2_ as a byproduct. Consequently, increased metabolic activity typically elevates blood CO_2_ concentration [22]. As pregnancy progresses and lactation begins, energy demands surge [23], necessitating accelerated glucose, fat, and protein metabolism [24]. This was supported by elevated blood TCO_2_ and related parameters, including pCO_2_, HCO_3_^−^, and BEecf, suggesting a heightened oxygen requirement to sustain increased metabolic activity postpartum. Despite this increased demand, we observed a decline in oxygen supply and transport parameters, especially for Hb concentration. This may be attributed to a reduced number of red blood cells during this period. Previous studies have consistently observed a significant decline in red blood cell count during the late periparturient period [25–27], suggesting a reduced oxygen transport capacity that leads to a relative negative oxygen balance characterized by decreased oxygen delivery and increased oxygen consumption, which may trigger systemic hypoxic stress. However, the hypoxic condition described in this study is not equivalent to hypoxemia caused by high-altitude exposure or respiratory failure. Instead, it represents a relative reduction in oxygen availability within the physiological range. The fact that arterial pO_2_ and sO_2_ did not show dramatic changes further supports this interpretation. One limitation of the present study is that blood samples were obtained from the coccygeal vein, which may not fully represent oxygen dynamics in specific organs or tissues. However, coccygeal blood sampling is widely used in dairy cow studies to reflect systemic physiological status. Future studies using more targeted sampling approaches may help to further elucidate tissue-specific oxygen metabolism during the transition period.

Previous studies have reported a significant upregulation of HIF-1α expression in the mammary gland [28], liver [29], and adipose tissue [30] after parturition, suggesting that major metabolic organs are under hypoxic stress. The decreased peripheral oxygen concentration observed in the current study confirmed this. These findings corroborate the hypothesis of negative oxygen balance during the transition period, with the decline in red blood cells and hemoglobin as key contributing factors. Hormonal fluctuations and periparturient stress likely contribute to this outcome. Reduced energy availability postpartum may inhibit Hb synthesis, lowering Hb concentrations and subsequently HCT [21]. Additionally, plasma volume expansion near parturition supports maternal–fetal nutrient flow but causes hemodilution, reducing oxygen-carrying capacity and contributing to hypoxemia [31]. Elevated estrogen levels suppress erythropoietin production, further diminishing HCT and Hb [32]. Rising cortisol levels during parturition exacerbate this effect by inhibiting erythropoiesis [33]. These combined factors indicate that postpartum dairy cows may experience metabolizable oxygen shortages, leading to hypoxia. The exact primary factors and molecular mechanisms behind the decrease in Hb and red blood cells during the perinatal period need to be further explored in future studies.

Hypoxia can negatively impact animal health and production performance [34]. Notably, Hb, HCT, and cO2 reached their lowest levels around d +21 postpartum, suggesting that this stage may represent the peak of hypoxia during the transition period. Therefore, HIF-1α at d +21 was used to classify cows according to their hypoxic status in current study to evaluate the effects of hypoxia on transition dairy cows. Our findings align with these observations, as cows with relative lower hypoxia exhibited better health and production performance throughout the transition period. Adequate oxygen supply supports nutrient availability in the rumen, promoting microbial activity, enhancing digestion, and increasing DMI [35]. Conversely, restricted oxygen availability limits ATP synthesis, crucial for milk production, thereby reducing lactation performance [36]. Lower ALT activity in less hypoxic cows suggests reduced liver stress and better liver function, correlating with lower BHBA levels. Hypoxia is also a recognized stressor that triggers systemic inflammation [37]. Acute-phase proteins such as HPT and SAA increased in response to hypoxic stress, indicating heightened inflammation in more hypoxic cows [38]. Additionally, lower MDA levels in less hypoxic cows reflect reduced oxidative stress [39]. The combined effects of inflammation and oxidative stress further diminish DMI, exacerbating negative energy balance and impairing early lactation performance [40]. These results indicate that ensuring an adequate supply of metabolizable oxygen for high-producing dairy cows is crucial for both their production performance and health. Future research should explore the full impact of relative hypoxia on periparturient cow health to develop strategies that optimize welfare and productivity during this critical period.

## CONCLUSION

The transition period in dairy cows involves significant physiological and metabolic changes, increasing oxygen demand. In the current study, we observed a postpartum decline in Hb and HCT levels, indicating a negative oxygen balance characterized by decreased oxygen delivery and increased oxygen consumption, potentially contributing to relative metabolic hypoxia and hypoxic stress, which associated with heightened liver damage, inflammation, and oxidative stress, ultimately impairing health and production performance. These findings highlight the importance of addressing metabolizable oxygen insufficiency during the transition period. Future research should focus on exploring strategies to mitigate hypoxic stress in periparturient dairy cows through targeted nutritional and management interventions to enhance welfare and productivity.

## Figures and Tables

**Table 1:** Ingredients and chemical composition of diet (% of DM)

Item	Prepartum	Postpartum
Ingredients		
Corn	18.8	22.0
Soybean meal	14.2	12.7
Flaked maize	-	8.21
Rapeseed meal	-	5.41
Sugar beet plup	-	5.17
Molasses	-	1.97
Corn silage	24.3	25.2
Alfalfa silage	-	3.28
Alfalfa hay	-	7.38
Oat hay	39.4	3.65
Fatty acid	-	1.27
Fatty acid calcium	-	0.28
Urea	-	0.37
Sodium bicarbonate	-	0.70
CaHPO_4_	0.30	0.41
NaCl	0.15	0.45
MgO	0.23	0.25
Stone powder	-	0.94
Anion salt1)	1.39	-
Premix2)	1.20	0.41
Composition		
CP	15.0	17.0
Starch	14.8	23.8
NDF	47.7	33.8
ADF	30.1	21.0
Crude ash	7.35	7.91
Ca	0.53	1.05
P	0.18	0.29
NEL (Mcal/kg)3)	1.51	1.76

1) Anion salt: −1,436 mEq/100 g.

2) Prepartum formulated to provide for one kilogram of premix: 464,286 IU vitamin A; 7,500 mg vitamin E; 209,184 IU vitamin D; 0.969 g Fe; 0.918 g Cu; 1.93 g Zn; 1.37 g Mn; 33 mg I; 27 mg Se; 27 mg Co. Postpartum formulated to provide for one kilogram of premix: 1,360,000 IU vitamin A; 360,000 IU vitamin D; 9,000 mg vitamin E; 3 g Cu; 18g Zn; 10.5g Mn; 88 mg Se; 185 mg I; 95 mg Co.

3) Calculated based on the Ministry of Agriculture [41].

**Table 2:** Comparison of lactation performance of cows with high (HHS) and low (LHS) levels of hypoxia-inducible factor 1α (HIF-1 α) expression

Items1)	Expression of HIF-1α		SEM	p-value		
	
	HHS	LHS		Group	Week	Group×Week
DMI (kg/d)						
Prepartum	20.1	20.2	0.97	0.10	<0.01	0.10
Postpartum	22.3	25.6	0.99	0.04	<0.01	0.08
Yield (kg/d)						
Milk	47.1	53.7	1.39	0.07	<0.01	0.05
Fat	1.71	1.82	0.11	0.45	0.04	0.48
Protein	1.47	1.54	0.03	0.10	0.79	0.10
ECM	49.3	53.4	1.58	0.09	0.25	0.59
Composition (%)						
Fat	3.64	3.39	0.15	0.04	<0.01	0.41
Protein	3.13	2.87	0.07	0.08	<0.01	0.07
Lactose	4.97	4.91	0.09	0.53	<0.01	0.49
TS	12.3	12.1	0.27	0.41	<0.01	0.48
SCC (×10^3^/mL)	350	532	90.0	0.59	0.68	0.56
MUN (mmol/L)	10.5	9.14	0.42	0.03	0.17	0.79

1) ECM = 0.3246×milk yield+13.86×milk fat yield+7.04×milk protein yield.

SEM, standard error of the mean; DMI, dry matter intake; ECM, energy-corrected milk; TS, total solids; SCC, somatic cell count; MUN, milk urea nitrogen.

**Table 3:** Comparison of arterial and venous blood gases in cows high (HHS) and low (LHS) levels of hypoxia-inducible factor 1α (HIF-1 α) expression

Items		Expression of HIF-1α		SEM	p-value		
	
		HHS	LHS		Group	Week	Group×Week
HIF-1α (ng/L)	V	1,552	1,191	88.6	<0.01	-	-
pH	A	7.45	7.46	0.01	0.75	0.01	0.27
	V	7.36	7.42	0.01	0.02	<0.01	0.02
Na^+^ (mmol/L)	A	141	141	0.28	0.62	<0.01	0.01
	V	141	141	0.34	0.54	<0.01	0.14
K^+^ (mmol/L)	A	4.10	4.04	0.04	0.28	<0.01	0.81
	V	4.46	4.47	0.06	0.91	<0.01	0.01
iCa (mmol/L)	A	1.19	1.18	0.02	0.51	<0.01	0.69
	V	1.22	1.22	0.02	0.96	<0.01	0.57
TCO_2_ (mmol/L)	A	27.5	28.4	0.70	0.37	<0.01	0.61
	V	30.6	29.6	0.58	0.20	<0.01	0.32
pCO_2_ (mmHg)	A	37.6	38.5	1.00	0.51	<0.01	0.99
	V	51.6	47.0	1.09	<0.01	<0.01	0.25
HCO_3_^−^ (mmol/L)	A	26.4	27.1	0.66	0.52	0.14	0.26
	V	28.1	29.0	0.58	0.29	<0.01	0.25
BEecf (mmol/L)	A	2.36	2.94	0.74	0.58	<0.01	0.50
	V	3.20	3.46	0.62	0.77	<0.01	0.12
HCT (%)	A	25.5	25.2	0.55	0.72	<0.01	0.33
	V	25.8	25.7	0.55	0.92	<0.01	0.23
Hb (g/dL)	A	8.67	8.59	0.19	0.76	<0.01	0.32
	V	8.77	8.78	0.17	0.95	<0.01	0.49
pO_2_ (mmHg)	A	143	156	7.20	0.22	0.14	0.79
	V	34.7	29.8	0.90	<0.01	0.34	0.04
sO_2_ (%)	A	99.0	99.3	0.13	0.19	0.50	0.20
	V	61.6	51.7	1.98	<0.01	0.49	0.01
cO_2_ (mL/dL)	A	12.4	12.3	0.27	0.90	<0.01	0.16
	V	7.78	6.37	0.24	<0.01	0.43	<0.01

V, vein; A, artery; HCT, hematocrit; Hb, hemoglobin; pO_2_, partial pressure of oxygen; sO_2_, oxygen saturation; cO_2_, concentration of oxygen = 0.003×pO_2_+1.34×Hb×sO_2_.

**Table 4:** Comparison of blood biochemical indicators in cows with high (HHS) and low (LHS) levels of hypoxia-inducible factor 1α (HIF-1 α) expression

Items^1)^	Expression of HIF-1α		SEM	p-value		
	
	HHS	LHS		Group	Week	Group×Week
ALT (U/L)	20.4	18.4	0.77	0.08	<0.01	0.10
AST (U/L)	99.8	100	8.21	0.98	<0.01	0.88
Total protein (g/L)	74.2	77.5	1.99	0.25	0.35	0.34
Albumin (g/L)	29.1	28.1	0.87	0.39	0.07	0.04
Globulin (g/L)	45.6	49.4	2.28	0.25	0.05	0.37
A/G	0.64	0.57	0.03	0.27	0.04	0.05
BUN (mmol/L)	5.74	5.08	0.27	0.09	<0.01	0.45
Glucose (mmol/L)	3.63	3.89	0.15	0.24	<0.01	0.35
BHBA (mmol/L)	521	439	20.8	0.01	<0.01	0.67
Cholesterol (mg/dL)	2.37	2.54	0.15	0.41	<0.01	0.15
Triglycerides (mmol/L)	0.17	0.16	0.01	0.82	<0.01	0.95
NEFA (μmol/L)	332	290	29.3	0.32	<0.01	0.26
SOD (U/mL)	120	139	5.43	0.08	<0.01	0.46
GPX (U/mL)	151	147	6.72	0.73	0.01	0.04
TAS (mmol/L)	0.38	0.41	0.02	0.24	<0.01	0.23
TOS (μmol/L)	7.22	7.25	0.24	0.45	<0.01	0.14
OSI	20.1	22.4	1.21	0.19	<0.01	0.37
MDA (nmol/mL)	2.29	1.90	0.12	0.07	<0.01	0.40
SAA (μg/L)	512	503	28.3	0.82	<0.01	0.48
Haptoglobin (μg/mL)	1,788	1,652	34.5	0.01	<0.01	0.06

ALT, alanine aminotransferase; AST, aspartate aminotransferase; A/G, albumin/globulin ratio; BUN, blood urea nitrogen; BHBA, β-hydroxybutyrate; NEFA, nonesterified fatty acids; SOD, sureroxide dismutase; GPX, glutathione peroxidase; TAS, total antioxidative capacity; TOS, total oxidative capacity; OSI, oxidative stress index; MDA, malondialdehyde; SAA, serum amyloid A.

## SUPPLEMENTARY MATERIAL

**Supplement 1.** Basic indicators of dairy cows in high and low HIF groups at +21d after calving.

**Supplement 2.** Dynamic changes of dry matter intake (DMI) and postpartum milk yield in 46 dairy cows.

**Supplement 3.** Dynamic changes of blood biochemistry in 46 dairy cows before and after calving at −21d, −7d, +7d, +21d, +35d, and +49d.
