# Supplementary material for: Longitudinal blood gas profiling reveals relative systemic hypoxia and its consequences for health and production in transition dairy cows
Source: Anim Biosci. 2026 Jun 1;39(9):250687. doi: 10.5713/ab.250687 (PMC13547630; doi:10.5713/ab.250687)
Supplement: Supplementary file 1 [file ab-250687-Supplementary-1.pdf]

Supplement 1. Basic indicators of dairy cows in high and low HIF groups at +21d after calving

1Items	Expression of HIF-1α		SEM	P-value
	HHS	LHS		
AVD , mL/dL	3.50	7.50	0.14	<0.01
Parity	1.90	1.70	0.09	0.34
Milk yield , kg	51.4	51.3	1.42	0.87
Milk effiency	2.10	2.20	0.46	0.54
Body condition	3.00	3.10	0.55	0.98

[1]AVD = Arteriovenous Oxygen Difference; Milk efficiency = Milk yield/DMI.
