# Supplementary material for: Longitudinal blood gas profiling reveals relative systemic hypoxia and its consequences for health and production in transition dairy cows
Source: Anim Biosci. 2026 Jun 1;39(9):250687. doi: 10.5713/ab.250687 (PMC13547630; doi:10.5713/ab.250687)
Supplement: Supplementary file 2 [file ab-250687-Supplementary-2.pdf]

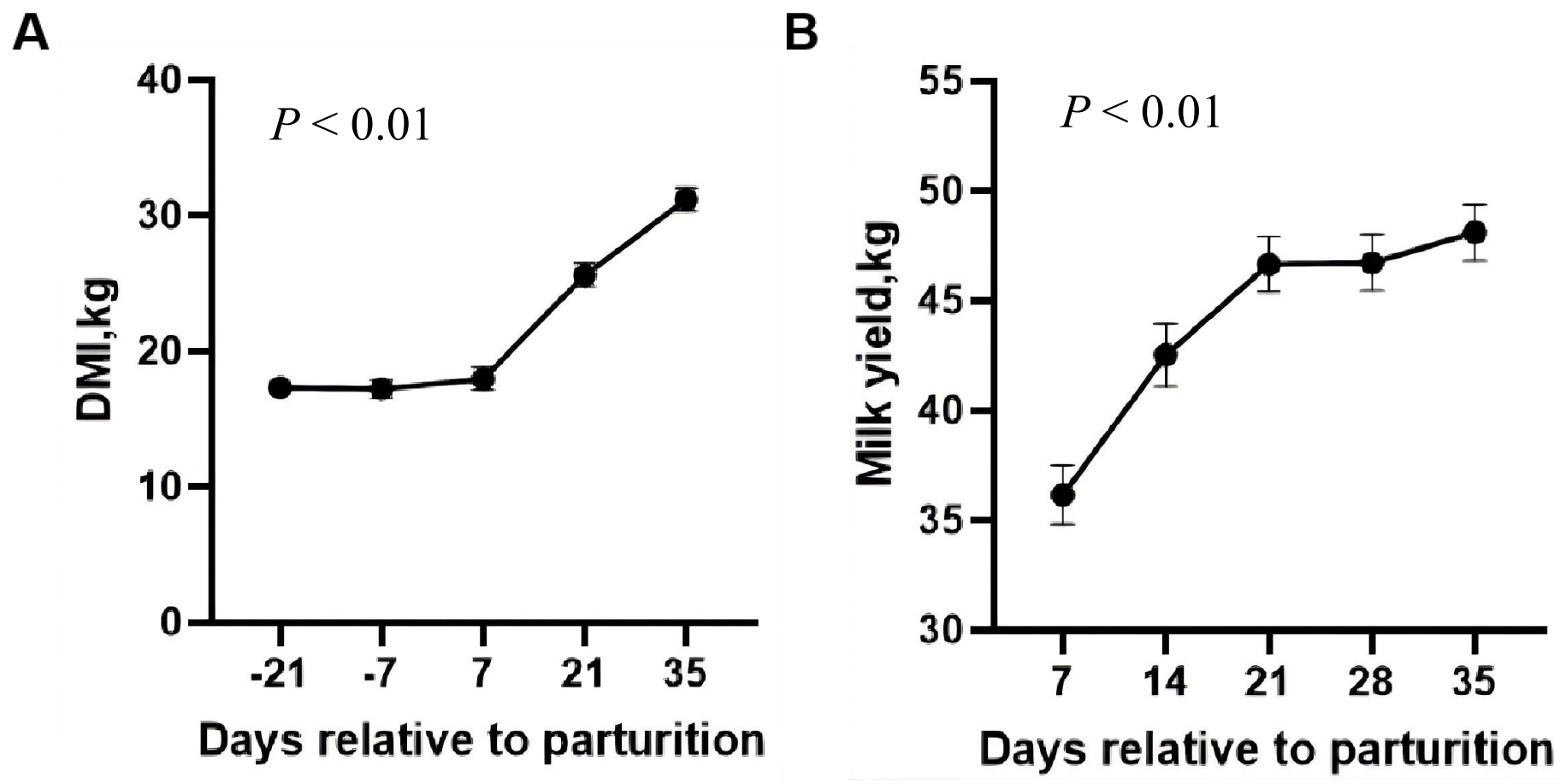

Supplement 2. Dynamic changes of dry matter intake (DMI) and postpartum milk yield in 46 dairy cows.
