# Supplementary material for: Longitudinal blood gas profiling reveals relative systemic hypoxia and its consequences for health and production in transition dairy cows
Source: Anim Biosci. 2026 Jun 1;39(9):250687. doi: 10.5713/ab.250687 (PMC13547630; doi:10.5713/ab.250687)
Supplement: Supplementary file 3 [file ab-250687-Supplementary-3.pdf]

Supplement 3. Dynamic changes of blood biochemistry in 46 dairy cows before and after calving at -21d, -7d, +7d, +21d, +35d, and +49d.

Items*	-21d	-7d	0d	+7d	+21d	+35d	+49d	SEM	P-value
ALT , U/L	23.8	21.3	19.4	18.5	16.1	22.2	25.9	0.77	<0.01
AST,U/L	77.3	89.0	105	119	101	97.8	99.5	4.75	<0.01
Total Protein , g/L	78.1	74.4	70.9	75.5	75.9	83.7	86.2	1.53	<0.01
Albumin , g/L	27.6	29.0	29.0	27.4	26.0	30.7	31.6	0.63	<0.01
Globulin , g/L	50.5	45.4	41.9	48.1	50.0	54.6	57.0	1.43	<0.01
A/G	0.55	0.64	0.69	0.57	0.52	0.56	0.55	0.04	<0.01
BUN , mmol/L	6.49	6.66	5.02	4.21	4.09	6.48	7.56	0.76	<0.01
BHBA， mmol/L	416	376	287	646	579	630	645	25.8	<0.01
Glucosde , mmol/L	3.62	3.78	4.97	3.26	3.10	4.95	5.71	0.79	<0.01
Cholesterol , mg/dL	2.46	2.11	1.67	2.20	3.47	6.9.0	8.07	0.77	<0.01
Triglyceride , mmol/L	0.26	0.27	0.10	0.10	0.10	1.69	2.33	0.86	0.04
NEFA , µmol/L	127	241	483	464	246	181	122	26.1	<0.01
TAS , mmol/L	0.32	0.33	—	0.40	0.35	—	—	0.86	0.02
TOS , µmol/L	7.54	6.34	—	5.87	8.20	—	—	0.20	＜0.01
OSI	24.2	20.5	—	14.9	24.4	—	—	0.96	＜0.01
SOD , U/mL	129	138	143	135	127	157	161	3.98	<0.01
GPX , g/L	161	156	142	132	141	155	164	5.43	<0.01
MDA , nmol/ml	0.81	2.01	—	2.00	3.29	—	—	0.14	＜0.01
SAA , mg/L	331	196	—	696	769	—	—	22.4	＜0.01
Haptoglobin , µg/mL	1,950	1,941	—	1,416	1,674	—	—	32.3	＜0.01

1NEFA = Nonesterified fatty acids; ALT = Alanine aminotransferase; AST = Aspartate aminotransferase; TAS = Total antioxidative capacity;TOS = Total oxidative capacity; MDA = Malondialdehyde; SOD = Sureroxide dismutase; GPX = Glutathione peroxidase; SAA = Serum amyloid A;
